# Supplementary material for: Association of rotating night shift work with tooth loss and severe periodontitis among permanent employees in Japan: a cross-sectional study
Source: PeerJ. 2024 Apr 16;12:e17253. doi: 10.7717/peerj.17253 (PMC11027908; doi:10.7717/peerj.17253)
Supplement: Supplemental Information 3 [file peerj-12-17253-s003.pdf]

ID	質問文	選択肢
SC1	あなたの性別をお知らせください。<br>Please indicate your gender.	1. 男性<br>2. 女性<br>3. その他<br>1.Man<br>2.Woman<br>3.Other
SC2	あなたの年齢をお知らせください。<br>Please provide your age.	(　　)歳<br>(　　) years
SC3	あなたの雇用形態は何ですか。<br>What is your employment status?	1. 常時雇用されている正規職員<br>2. 派遣職員・契約職員・嘱託職員<br>3. 臨時雇用・パート・アルバイト<br>4. その他<br>1. Permanent employees<br>2. Fixed-term employees<br>3. Short-term employees<br>4. Others

ここではあなたの現在の仕事や経歴についてうかがいます。

We would like to inquire about your current job and career history here.

ID	質問文	選択肢
SC7	あなたの仕事は､次のどれにあてはまりますか。<br>Which of the following best describes your occupation?	1. 管理的職業<br>2. 専門的・技術的職業<br>3. 事務的職業<br>4. 販売の職業<br>5. サービスの職業<br>6. 保安の職業<br>7. 農林漁業の職業<br>8. 生産工程の職業<br>9. 輸送・機械運転の職業<br>10. 建設・採掘の職業<br>11. 運搬・清掃・包装等の職業<br>12. その他<br>1=Administrative and managerial workers<br>2=Professional and engineering workers<br>3=Clerical workers<br>4=Sales workers<br>5=Service workers<br>6=Security workers

		7=Agriculture, forestry and fishery workers<br>8=Manufacturing process workers<br>9=Transport and machine operation workers<br>10=Construction and mining workers<br>11=Carrying, cleaning, packaging, and related workers<br>12=Workers not classifiable by occupation
SC8	現在、あなたは何らかの役職についていますか。<br>Do you currently hold any position or title?	1.役職はない<br>2.監督・職長・班長・組長<br>3.係長・係長相当職<br>4.課長・課長相当職<br>5.部長・部長相当職<br>6.社長・重役・役員・理事<br>7.その他<br>1=No position<br>2=Supervisor<br>3=Section chiefs<br>4=Section manager<br>5=Department managers<br>6=President/executive/officer/director<br>7=Other

ID	質問文	選択肢
Q3	あなたは過去 7 日間に、合計で何時間働きましたか？<br>(97 時間より多かったら、97 と入れてください)<br>How many hours does your employer expect you to work in a typical 7-day week?<br>(If it varies, estimate the average. If more than 97, enter 97.)	(　　)時間<br>(　　) hours

ID	質問文	選択肢
Q6	交代制勤務で、あなたが夜間シフトに入った年数の合計(日勤のほかに、ひと月に 3 回以上夜勤を担当した年数)をお答えください。<br>What is the total number of years during which you worked rotating night shifts (at least 3 nights/month in addition to days or evenings in that month)?	1. 1 回もない<br>2. 1〜2 年<br>3. 3〜5 年<br>4. 6〜9 年<br>5. 10〜14 年<br>6. 15〜19 年<br>7. 20〜29 年<br>8. 30 年以上<br>1=Never<br>2=1–2 years<br>3=3–5 years<br>4=6–9 years<br>5=10–14 years<br>6=15–19 years<br>7=20–29 years<br>8=30 years or more

ID	質問文	選択肢
Q9	あなたはこれまでの質問に対して、正確に答えましたか？<br>Did you answer the previous questions accurately?	1. いいえ<br>2. はい、正確に答えました。<br>1. No<br>2. Yes, I answered them accurately

あなたの現在の健康状態や生活習慣についてお聞きします。

We would like to inquire about your current health status and lifestyle habits.

ID	質問文	選択肢
Q10	次の選択肢から、これまでにあなたが診断された、もしくは現在治療を受けているものを選んで下さい。複数選択が可能です。病気になったことがない方は、「ない」を選んでください。<br>Please select from the following options any conditions that you have been diagnosed with or are currently receiving treatment for. Multiple selections are possible. If you have never been ill, please select "None."	1. ない<br>6. 糖尿病<br>8. 狭心症・心筋梗塞<br>9. 脳卒中(脳梗塞や脳出血)<br>10. その他の心臓病<br>1. None<br>6. Diabetes<br>8. Coronary Heart Disease<br>9. Stroke<br>10. Other heart diseases
Q11	あなたは紙巻きタバコを吸っていますか。<br>加熱式タバコや電子タバコは除外してください。<br>Do you smoke conventional cigarettes? Please exclude heated tobacco products or electronic cigarettes.	1. これまで一度も吸ったことがない。<br>2. 1 回以上吸ってみたことがあるが、習慣的には使用しなかった。<br>3. 以前は習慣的に吸っていたが、今は止めている。<br>4. 時々吸う日がある。<br>5. ほとんど毎日吸っている。<br>1.Never smoked before.<br>2.Tried smoking once or more, but not habitually.

		3.Used to smoke habitually in the past, but quit.<br>4.Smoke occasionally.<br>5.Smoke almost every day.
Q12	あなたは加熱式タバコや電子タバコを吸っていますか。<br>紙巻きタバコは除外してください。<br>Do you use heated tobacco products or electronic cigarettes?<br>Please exclude conventional cigarettes.	1. これまで一度も吸ったことがない。<br>2. 1 回以上吸ってみたことがあるが、習慣的には使用しなかった。<br>3. 以前は習慣的に吸っていたが、今は止めている。<br>4. 時々吸う日がある。<br>5. ほとんど毎日吸っている。<br>1=Never smoked<br>2=Smoked once or more, but not habitually<br>3=Used to smoke habitually, but quit<br>4=Smoke occasionally<br>5=Smoke almost every day
Q13	過去 1 年間に、どのくらいアルコール(ビール、発泡酒、チューハイ、ウィスキー、焼酎、日本酒、ワインなど)を飲みましたか。<br>In the past year, how often have you consumed alcohol (beer, happoshu, chuhai, whiskey, shochu, sake, wine, etc.)?	1. 毎日<br>2. 1 週間に 5~6 日<br>3. 1 週間に 3~4 日<br>4. 1 週間に 1~2 日<br>5. 1 ヵ月に数回程度

		6. ほとんど飲まない<br>7. 飲めない<br>1=Every day<br>2=5-6 days a week<br>3=3-4 days a week<br>4=1-2 days a week<br>5=A few times a month<br>6=Almost never<br>7=Cannot drink
Q14	あなたはどのくらいの頻度で、歯科医院で予防的な定期管理をうけていますか。<br>治療による受診は含まないで下さい。<br>How often do you receive preventive regular check-ups at a dental clinic? (Please do not include visits for treatment)	1. うけていない<br>2. 約半年に 1 回<br>3. 約 1 年に 1 回<br>4. 2～3 年に 1 回<br>1=I don't receive them<br>2=About every six months<br>3=About once a year<br>4=Every 2-3 years
Q15	どれくらいの頻度で歯みがきをしますか。<br>How many times a day do you brush your teeth?	1. 1 日 3 回以上<br>2. 1 日 2 回<br>3. 1 日 1 回<br>4. 数日に 1 回<br>5. みがかない<br>1=Three times or more per day<br>2=Twice a day<br>3=Once a day<br>4=Every few days

		5=I don't brush my teeth.
Q18	現在のあなたの生えている歯の本数はいくつですか。<br>親知らずを除くと､歯は全部で 28 本あります。<br>親知らずを含めると､歯は全部で 32 本あります。<br>インプラント､入れ歯､ブリッジは数に入れないでください。<br>How many natural teeth do you currently have? (excluding wisdom teeth, there are 28 teeth. Including wisdom teeth, there are 32 teeth. Please do not include dental implants, dentures, and dental bridges in your count.)	[ ]本<br>[ ] teeth

ID	質問文	選択肢
Q22	歯ぐきの病気にかかっているかもしれないと思いますか？<br>Do you think you might have gum disease?	1. はい<br>2. いいえ<br>3. 答えたくない<br>4. わからない<br>1=Yes<br>2=No<br>3=Prefer not to answer<br>4=Don't know
Q23	自然と歯がぐらつくようになったことはありますか？<br>(怪我によるものは除きます。)<br>Have you ever had any teeth become loose on their own, without an injury?	1. はい<br>2. いいえ<br>3. 答えたくない<br>4. わからない<br>1=Yes<br>2=No<br>3=Prefer not to answer<br>4=Don't know
Q24	歯医者あるいは歯科衛生士から「歯のまわりの骨が失われている」と言われたことがありますか？<br>Have you ever been told by a dental professional that you lost bone around your teeth?	1. はい<br>2. いいえ<br>3. 答えたくない<br>4. わからない<br>1=Yes<br>2=No

		3=Prefer not to answer<br>4=Don't know
Q25	ここ 3 ヵ月間で､歯ぐきから血が出たことはありますか？<br>During the past three months, have you had bleeding gums?	1. まったくない<br>2. ほとんどない<br>3. 時々<br>4. しばしば<br>5. いつも<br>1=Never<br>2=Hardly ever<br>3=Sometimes<br>4=Fairly often<br>5=Very often

Q26　過去１ヵ月の間､どのくらいの頻度で次のことがありましたか。

The following questions ask about how you have been feeling during the past 30 days. For each question, please circle the number that best describes how often you had this feeling. During the past 30 days, about how often did you feel …

ID	質問文	選択肢
	神経過敏に感じましたか。<br>…nervous?	1. 全くない<br>2. 少しだけ<br>3. ときどき<br>4. たいてい<br>5. いつも<br>1=None of the time<br>2=A little of the time<br>3=Some of the time<br>4=Most of the time<br>5=All of the time
	絶望的だと感じましたか。<br>…hopeless?	
	そわそわ､落ち着きがなく感じましたか。<br>…restless or fidgety?	
	気分が沈んで､何が起こっても気が晴れないように感じましたか。<br>…so depressed that nothing could cheer you up?	
	何をするのも骨折りだと感じましたか。<br>…that everything was an effort?	
	自分は価値がない人間だと感じましたか。<br>…worthless?	

Q27　以下の内容についてお答えください。

Please respond to the following content.

ID	質問文	選択肢
	自分には人との付き合いがないと感じることがありますか。<br>How often do you feel that you lack companionship?	1. 決してない<br>2. ほとんどない<br>3. 時々ある<br>4. 常にある<br>1=never<br>2=rarely<br>3=sometimes<br>4=always
	自分は取り残されていると感じることがありますか。<br>How often do you feel close to people?	
	自分は他の人たちから孤立していると感じることはありますか。<br>How often do you feel isolated from others?	

経済状況が健康を損なうという研究報告が多く報告されています。下記の質問は、全ての人が健康を維持していくための貴重なデータとなるものです。できるだけお答えください。

The economic situation has been reported to adversely affect health in many research studies. The following questions are valuable data for maintaining health for everyone. Please answer as much as possible.

ID	質問文	選択肢
Q28	あなたの婚姻状況は、次のうちのどれにあてはまりますか。<br>What is your marital status?	1. 結婚している<br>2. 離婚して、再婚はしていない<br>3. 離婚して、再婚している<br>4. 未婚である<br>5. 死別した<br>6. その他<br>1=Married<br>2=Divorced and not remarried<br>3=Divorced and remarried<br>4=Unmarried<br>5=Widowed<br>6=Other
Q29	現在、一緒に住んでいる方はあなたを含めて何人ですか。<br>一人暮らしの場合は 1 を入力してください。<br>How many people, including yourself, currently live with you? If you live alone, please enter "1."	あなたを含めて(　　　)人<br>Number of people, including yourself: ______.
Q31	あなたと一緒に住んでいる同居者を含んだ世帯全体の合計の 1 年間の収入は税込みでいくら見込めますか。	年間(　　　)万円

	株式配当、臨時収入、副収入などすべての収入を含んだものをお答えください。<br>What is the estimated total annual income before tax for the entire household, including yourself and any other cohabitants? Please include all sources of income such as stock dividends, occasional income, secondary income, etc.	Annual income: ______ million yen.
Q34	あなたの最終学歴を教えてください。<br>Please indicate your highest level of education completed	1. 中学校 中退<br>2. 中学校 卒業<br>3. 高校 中退<br>4. 高校 卒業<br>5. 専門学校 中退<br>6. 専門学校 卒業<br>7. 短期大学／高等専門学校 中退<br>8. 短期大学／高等専門学校 卒業<br>9. 大学(4 年制) 卒業<br>10. 大学(4 年制) 中退<br>11. 大学（6 年制）卒業<br>12. 大学（6 年制）中退<br>13. 大学院修士課程 修了<br>14. 大学院修士課程 中退<br>15. 大学院博士課程 修了<br>16. 大学院博士課程 中退<br>1=Junior high school, did not graduate<br>2=Junior high school, graduated<br>3=High school, did not graduate<br>4=High school, graduated

		5=Professional training college, did not graduate<br>6=Professional training college, graduated<br>7=Junior college/technical college, did not graduate<br>8=Junior college/technical college, graduated<br>9=University (4-year program), graduated<br>10=University (4-year program), did not graduate<br>11=University (6-year program), graduated<br>12=University (6-year program), did not graduate<br>13=Master's program, completed<br>14=Master's program, did not complete<br>15=Doctoral program, completed<br>16=Doctoral program, did not complete

ID	質問文	選択肢
Q35	あなたはこれまでの質問に対して、正確に答えましたか？<br>Did you answer the previous questions accurately?	1. はい、正確に答えました。<br>2. いいえ<br>1. Yes, I answered them accurately<br>2. No
